# Prognostic impact of peritonitis in hemodialysis patients: A national-wide longitudinal study in Taiwan

**DOI:** 10.1371/journal.pone.0173710

**Published:** 2017-03-16

**Authors:** Yueh-An Lu, Kun-Hua Tu, Cheng-Chia Lee, Patricia W. Wu, Chee-Jen Chang, Ya-Chung Tian, Chih-Wei Yang, Pao-Hsien Chu

**Affiliations:** 1 Kidney Research Center, Department of Nephrology, Linkou Chang Gung Memorial Hospital, Taipei, Taiwan; 2 Department of Medicine, Chang Gung University, Taoyuan, Taiwan; 3 Graduate Institute of Clinical Medical Sciences, College of Medicine, Chang Gung University, Taoyuan, Taiwan; 4 Department of Radiology, Chang Gung Memorial Hospital, Linkou, Taiwan; 5 Graduate Institute of Clinical Medicine, Chang Gung University, Tao-Yuan, Taiwan; 6 Research Services Center for Health Information, Chang Gung University, Tao-Yuan, Taiwan; 7 Clinical Informatics and Medical Statistics Research Center, Chang Gung University, Tao-Yuan, Taiwan; 8 Department of Cardiology, Chang Gung Memorial Hospital, Chang Gung University College of Medicine, Taipei, Taiwan; 9 Healthcare Center, Chang Gung Memorial Hospital, Chang Gung University College of Medicine, Taipei, Taiwan; 10 Heart Failure Center, Chang Gung Memorial Hospital, Chang Gung University College of Medicine, Taipei, Taiwan; The University of Tokyo, JAPAN

## Abstract

**Background:**

Peritonitis has been independently associated with increased morbidity and mortality in peritoneal dialysis patients. However, there are few reports on peritonitis in hemodialysis patients. We aim at investigating both the risk profiles and prognostic impact of peritonitis in hemodialysis patients.

**Methods:**

This nation-wide longitudinal study uses claims data obtained from the Taiwan National Health Insurance Research Database. A total of 80,733 incident hemodialysis patients of age ≥ 20 years without a history of peritonitis were identified between January 1, 1998 and December 31, 2009. Predictors of peritonitis events were estimated using Cox proportional hazard models. Time-dependent Cox proportional hazard models were used to estimate hazard ratio for mortality attributed to peritonitis exposure.

**Results:**

Of 80,733 incident hemodialysis patients over a 13-year study period, peritonitis was diagnosed in 935 (1.16%), yielding an incidence rate of 2.91 per 1000 person-years. Female gender, liver cirrhosis and polycystic kidney disease were three of the most significant factors for peritonitis in both non-diabetic and diabetic hemodialysis patients. The cumulative survival rate of patients with peritonitis was 38.8% at 1 year and 10.1% at 5 years. A time-dependent Cox multivariate analysis showed that peritonitis had significantly increased hazard ratio for all cause mortality. Additionally, the risk of mortality remained significantly higher for non-diabetic hemodialysis patients that experienced peritonitis.

**Conclusions:**

The risk of peritonitis in hemodialysis patients is higher in female gender, liver cirrhosis and polycystic kidney disease. Although peritonitis is a rare condition, it is associated with significantly poorer outcome in hemodialysis patients.

## Introduction

The rapidly expanding global population of dialysis patients represents an important public health challenge not only in Taiwan, but all around the world [1–4]. At the end of 2013, there were over 70,000 patients living with end-stage renal disease (ESRD) and treated with either hemodialysis (HD) or peritoneal dialysis (PD) in Taiwan [5]. Recent published data from the international comparisons of the United States Renal Data System (USRDS) also revealed that Taiwan continues to report the highest prevalence (3,138 per million population) of treated ESRD [2]. Notably, individuals with ESRD have an increased risk of mortality and hospitalization [6]. Following cardiovascular disease, infections are the second most likely cause of hospitalization and mortality in dialysis patients [2, 6]. It has been established that disturbances in both innate and adaptive immune systems significantly contribute to this susceptibility [7–9]. The leading causes of infection-related hospitalization in HD patient were infection of central venous catheters, blood stream infection and pulmonary infection. In addition, peritonitis and other intra-abdominal infections cause 6.7~12.2% of infection events in HD patients [10, 11].

Peritonitis is caused by bacterial invasion of the peritoneal cavity. Patients often present with abdominal pain, ascites formation, systemic inflammation response syndrome (SIRS) and even multi-organ failure. Primary peritonitis is usually caused by bacterial translocation from the gut in patients with massive ascites. Secondary peritonitis is often related to hollow organ perforation, acute cholecystitis, mesenteric ischemia, and pyonephrosis [12]. Peritonitis is a well-known direct cause of mortality in PD patients, reported in around 1–6% in different studies [13, 14]. In addition, many studies have also explored the impact of peritonitis on the clinical outcome of PD patients [13, 15, 16]. It has been reported that peritonitis is a “contributing factor” to death in around 15% of deaths on PD [16], and that peritonitis rate is a significant predictor for mortality in non-diabetic patients and patients over the age of 60 [13]. However, few reports in the literature focus on peritonitis in HD patients. We hypothesized that, like PD patients, incident peritonitis is a significant factor for poor prognosis in HD patients. Because of the low incidence of peritonitis in HD cohorts, we analyzed datasets from the Taiwan National Health Insurance Research Database (NHIRD). The aim of this longitudinal cohort study is to investigate the risk profiles that determine the development of peritonitis in HD patients, and to investigate the prognostic impact of peritonitis in HD patients.

## Materials and methods

### Data source

This national-wide longitudinal cohort study was conducted by analyzing data from the Taiwan NHIRD. The National Health Insurance program of Taiwan has provided compulsory universal health insurance in Taiwan since 1995 and covered more than 99.6% of citizens and over 90% of hospitals. Therefore, NHIRD contains detailed healthcare data and offers researchers encrypted datasets including each patient’s gender, date of birth, date and dosage of prescriptions, procedure charge codes, the International Classification of Diseases, Ninth Revision, Clinical Modification (ICD-9-CM) diagnostic codes and outcome. This study was approved by the institutional review board of Chang Gung Memorial Hospital (approval number: 102-4175B), and the need for individual consent was waived because all personal information was de-identified in the encrypted datasets before being released to the public for medical research.

### Patient selection

**Fig 1:**
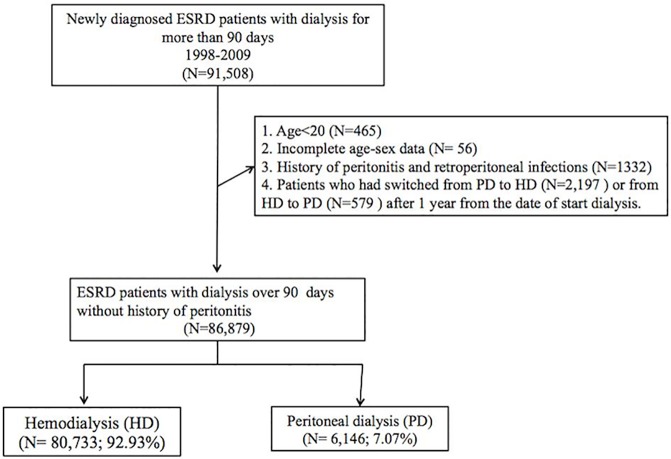
Flow diagram of patient enrollment.

A flow chart of the enrollment process of this study cohort is shown in Fig 1. Using data from NHIRD, we identified 91,508 patients who had newly diagnosed ESRD and undergone regular dialysis (either PD or HD) for at least 90 days in the outpatient department between January 1, 1998 and December 31, 2009. The index date was defined as the date 90 days after dialysis initiation. We excluded patients that were younger than 20 years old (N = 465) or had incomplete sex-age data at the date of dialysis initiation (N = 56). Patients with a history of peritonitis or retroperitoneal infections before dialysis initiation (N = 1,332) were also excluded. Some HD patients may have received PD initially and later switched to HD because of technique failure. Because predictors identified from this population might related to PD instead of HD, 2,197 patients who switched from PD to HD and 579 patients who switched from HD to PD were excluded to avoid confounding a potential impact of one modality to another. The remaining 86,879 patients were then assigned to either HD group or PD group, according to their modality on day 90. We finally analyzed a study population of 80,733 incident HD patients without any history of peritonitis.

### Definition of peritonitis, comorbid variables, and outcome

The demographic results of age and sex were recorded from NHIRD at the date of dialysis initiation. Peritonitis was defined by ICD-9-CM diagnostic code 567.x in at least one outpatient visit or discharge medical record. Diabetic mellitus (DM), hypertension (HTN), and hyperlipidemia were defined by ICD-9-CM diagnostic codes 401–405, 250, and 272 in patients who used medication for more than 90 days per year. Myocardial infraction (MI) and stroke were defined as disease diagnosed on previous hospitalization using diagnostic codes 410–414 and 431–434, 436. Other comorbidities were defined as diagnostic codes in admission or at least 3 ambulatory outpatient visits for heart failure (ICD-9-CM 428), atrial fibrillation (ICD-9-CM 427.31), liver cirrhosis (ICD-9-CM 571.5), connective tissue disease (ICD-9-CM 710, 714, 728), malignancy (ICD-9-CM 140–208) and polycystic kidney disease (ICD-9-CM 573.12). All patients were followed from the initiation of dialysis to the primary end point, namely, the diagnosis of peritonitis, death, or until December 31, 2010.

### Statistical analysis

The demographic results were summarized using descriptive statistics. Continuous data were expressed as the mean ± standard deviation (SD) and categorical data were expressed as numbers or percentages (%) of each item. The Cox proportional hazards model was used to estimate the risk of peritonitis. Hazard ratios (HRs) and 95% confidence intervals (CIs) were derived from Cox proportional hazards models. We stratified patients to diabetic and non-diabetic groups when evaluating risk factors of peritonitis since they had different clinical characteristics and outcomes after entering ESRD. We further used Cox proportional hazards model to identify factors determining patient mortality. Because peritonitis may develop during the follow-up period, we treated peritonitis exposure as a time-dependent covariate for survival analysis. Patient survival curves and survival probabilities in patients with or without peritonitis exposure were generated according to the Kaplan–Meier method. Differences in the survival curves between two groups were evaluated using the log rank test. A P value of < 0.05 was considered statistically significant. This study used statistical analysis software (SAS, version 9.4) for data analysis.

## Results

### Incidence of peritonitis

Over a 13-year study period, a total of 80,733 incident HD patients without a history of peritonitis were enrolled. Table 1 lists the features of the study population. The mean age of patients was 61.9 ± 13.6 years; 50.5% of patients were female, and 41.2% of patients had prevalent DM. Nine hundred thirty-five (1.16%) patients experienced event of peritonitis during a cumulative follow-up period of 320,926 patient-years, with an incidence rate of approximately 2.91 per 1000 person-years. The median (interquartile range) time from the initiation of dialysis to event of peritonitis was 3.2 (1.5–5.8) years.

**Table 1:** Baseline characteristics of the study cohort (N = 80,733).

	Patient number	Percentage
**Sex _Female**	40,748	(50.5%)
**Hypertension**	47,481	(58.8%)
**Diabetes mellitus**	33,241	(41.2%)
**Hyperlipidemia**	16,080	(19.9%)
**Myocardial infraction**	21,093	(26.1%)
**Stroke**	10,506	(13.0%)
**Heart failure**	23,675	(29.3%)
**Atrial fibrillation**	3,328	(4.1%)
**Liver cirrhosis**	4,347	(5.4%)
**Connective tissue disease**	7,669	(9.5%)
**Malignancy**	8,650	(10.7%)
**Polycystic kidney disease**	1,130	(1.4%)

### Risk factors of peritonitis

Table 2 and Table 3 summarized the univariate and multivariate Cox regression analysis for risk factors of peritonitis, respectively. On univariate analysis, age, female sex, liver cirrhosis, connective tissue disease, malignancy and PKD were associated with an increased risk for peritonitis among all HD patients. DM was associated with a decreased risk for peritonitis among all HD patients. These associations remained significant on multivariate analysis. HTN was associated with a decreased risk with borderline significance for peritonitis only on multivariate analysis of all HD patients.

**Table 2:** Predictors of peritonitis in incident HD patient by using cox proportional hazard model (simple regression).

Group	All			Non-DM			DM		
	HR	95%CI	*p*-value	HR	95%CI	*p*-value	HR	95%CI	*p*-value
**Age**	1.01	1.01–1.02	< .0001	1.02	1.01–1.02	< .0001	1.01	1.00–1.02	0.067
**Female gender**	1.24	1.09–1.41	0.001	1.18	1.02–1.37	0.031	1.37	1.06–1.77	0.015
**Hypertension**	0.91	0.80–1.04	0.164	0.97	0.83–1.15	0.741	1.10	0.79–1.53	0.583
**Diabetes mellitus**	0.78	0.67–0.91	0.001						
**Hyperlipidemia**	0.94	0.78–1.14	0.536	1.02	0.74–1.41	0.907	1.10	0.85–1.43	0.470
**Myocardial infraction**	1.11	0.95–1.30	0.197	1.23	1.00–1.51	0.050	1.13	0.87–1.46	0.379
**Stroke**	1.04	0.84–1.31	0.703	1.14	0.83–1.57	0.409	1.10	0.80–1.52	0.563
**Heart failure**	1.16	1.00–1.35	0.056	1.28	1.06–1.55	0.012	1.16	0.90–1.50	0.261
**Atrial fibrillation**	1.56	1.12–2.16	0.009	1.59	1.06–2.39	0.026	1.57	0.90–2.74	0.116
**Liver cirrhosis**	3.17	2.5–3.90	< .0001	3.36	2.63–4.31	< .0001	3.00	2.07–4.33	< .0001
**Connective tissue disease**	1.44	1.16–1.78	0.001	1.45	1.12–1.89	0.005	1.49	1.02–2.16	0.038
**Malignancy**	1.71	1.41–2.08	< .0001	1.76	1.40–2.20	< .0001	1.56	1.06–2.30	0.025
**Polycystic kidney disease**	1.73	1.15–2.62	0.009	1.50	0.96–2.35	0.073	3.94	1.26–12.31	0.018

**Table 3:** Predictors of peritonitis in incident HD patient by using cox proportional hazard model (multiple regression).

Group	All			Non-DM			DM		
	HR	95%CI	*p*-value	HR	95%CI	*p*-value	HR	95%CI	*p*-value
**Age**	1.01	1.01–1.02	< .0001	1.01	1.01–1.02	< .0001	1.01	0.99–1.02	0.325
**Female gender**	1.25	1.09–1.42	0.001	1.21	1.04–1.41	0.016	1.38	1.06–1.79	0.016
**Hypertension**	0.86	0.74–1.00	0.048	0.82	0.69–0.98	0.031	1.01	0.72–1.43	0.941
**Diabetes mellitus**	0.75	0.63–0.89	0.001						
**Hyperlipidemia**	1.09	0.88–1.33	0.435	1.04	0.74–1.44	0.839	1.10	0.85–1.44	0.465
**Myocardial infraction**	1.04	0.88–1.24	0.634	1.05	0.84–1.31	0.687	1.04	0.79–1.38	0.771
**Stroke**	1.06	0.84–1.33	0.649	1.05	0.76–1.45	0.772	1.06	0.77–1.48	0.713
**Heart failure**	1.11	0.94–1.30	0.235	1.13	0.92–1.38	0.243	1.06	0.81–1.40	0.666
**Atrial fibrillation**	1.32	0.94–1.86	0.105	1.29	0.85–1.97	0.233	1.40	0.79–2.48	0.248
**Liver cirrhosis**	3.16	2.56–3.89	< .0001	3.21	2.50–4.12	< .0001	3.08	2.12–4.49	< .0001
**Connective tissue disease**	1.40	1.13–1.73	0.002	1.42	1.09–1.84	0.010	1.39	0.95–2.02	0.087
**Malignancy**	1.46	1.19–1.77	<0.001	1.50	1.19–1.89	0.001	1.34	0.90–1.99	0.149
**Polycystic kidney disease**	1.73	1.14–2.63	0.010	1.60	1.02–2.50	0.040	4.06	1.30–12.72	0.016

Multiple Regression: adjusted the variables listed in Table 1.

Since the proportion of diabetic patients has increased over time (around 10% in 1998 to 30% in 2009) and they had different clinical characteristics, we stratified patients by the presence or absence of DM and analyzed separately for risk profiles that determine the development of peritonitis. In non-diabetic HD patients, the multivariate analysis demonstrated that factors independently associated with increased HR for event of peritonitis were as follows: age (adjusted HR per year = 1.01, 95% CI = 1.74–2.68, P<0.001), female sex (adjusted HR = 1.21 95% CI = 1.04–1.41, P = 0.016), liver cirrhosis (adjusted HR = 3.21, 95% CI = 2.50–4.12, P<0.001), connective tissue disease (adjusted HR = 1.42, 95% CI = 1.09–1.84, P = 0.01), malignancy (adjusted HR = 1.50, 95% CI = 1.19–1.89, P = 0.001) and PKD (adjusted HR = 1.60, 95% CI = 1.02–2.50, P = 0.04). However, HTN (adjusted HR = 0.82, 95% CI = 0.69–0.98, P<0.031) was associated with a decreased risk of peritonitis in non-diabetic HD patients. In diabetic HD patients, multivariable analysis revealed that female sex (adjusted HR = 1.38, 95% CI = 1.06–1.79, P<0.016), liver cirrhosis (adjusted HR = 3.08, 95% = CI 2.12–4.49, P<0.001), and PKD (adjusted HR = 4.06, 95% CI = 1.30–12.72, P = 0.016) were associated with higher risk of peritonitis.

### Prognostic impact of peritonitis

**Fig 2:**
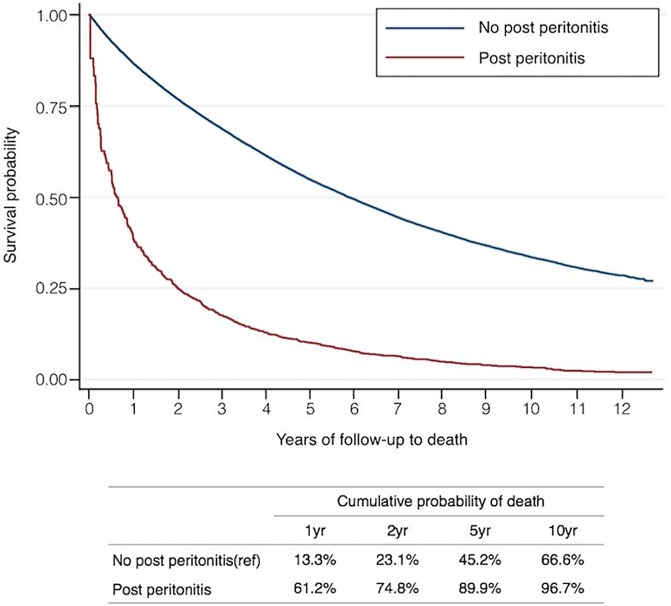
Kaplan–Meier survival curves for HD patients according to having peritonitis or not.

Fig 2 shows the Kaplan-Meier survival curves for HD patients according to peritonitis status. The cumulative survival rate of HD patients who experienced peritonitis was 38.8% at 1 year, 25.2% at 2 years, and 10.1% at 5 years. In comparison, the cumulative survival rate of HD patients who did not experience peritonitis was 86.7% at 1 year, 76.9% at 2 years, and 54.8% at 5 years. There was a significant difference in survival between the patient groups (log rank, P<0.001). In the multivariate Cox proportional hazard model using time-dependent covariate for peritonitis exposure, the adjusted HRs (95% CI) for mortality was 2.86 (2.63–3.12) (Table 4). We also compared the HRs for mortality between patients in the presence or absence of preexisting DM. Peritonitis exposure was still associated with higher risk of mortality in non-diabetic HD patients (adjusted HR = 3.09, 95% CI = 2.80–3.42, P<0.001) and the magnitude of increased HR was even higher than that in diabetic HD patients (adjusted HR = 2.37, 95% CI = 2.00–2.81, P<0.001). Additionally, age, myocardial infarction, stroke, heart failure, atrial fibrillation, liver cirrhosis and malignancy were all significantly related to an increased mortality risk. Hypertension and hyperlipidemia, by contrast, were associated with survival advantage.

**Table 4:** Factors that influence mortality in incident HD patient by using time-dependent covariate for peritonitis exposure (Multiple regression).

Group	All			Non-DM			DM		
	HR	95%CI	*p*-value	HR	95%CI	*p*-value	HR	95%CI	*p*-value
**No post peritonitis(ref)**	1			1			1		
**Post peritonitis**	2.86	2.63–3.12	<0.001	3.09	2.80–3.42	<0.001	2.37	2.00–2.81	<0.001
**Age**	1.04	1.04–1.04	<0.001	1.04	1.04–1.04	<0.001	1.03	1.03–1.03	<0.001
**Sex_Male**	1.16	1.14–1.19	<0.001	1.23	1.20–1.27	<0.001	1.02	0.99–1.06	0.134
**Hypertension**	0.80	0.78–0.82	<0.001	0.78	0.76–0.80	<0.001	0.81	0.78–0.85	<0.001
**Diabetes mellitus**	1.48	1.44–1.51	<0.001	-	-		-	-	
**Hyperlipidemia**	0.86	0.84–0.89	<0.001	0.82	0.77–0.87	<0.001	0.86	0.83–0.89	<0.001
**Myocardial infraction**	1.25	1.22–1.28	<0.001	1.29	1.25–1.34	<0.001	1.20	1.16–1.24	<0.001
**Stroke**	1.50	1.46–1.55	<0.001	1.71	1.64–1.78	<0.001	1.38	1.33–1.43	<0.001
**Heart failure**	1.32	1.29–1.35	<0.001	1.37	1.33–1.42	<0.001	1.24	1.20–1.28	<0.001
**Atrial fibrillation**	1.22	1.17–1.28	<0.001	1.36	1.28–1.44	<0.001	1.19	1.11–1.27	<0.001
**Liver cirrhosis**	1.74	1.68–1.81	<0.001	1.92	1.82–2.02	<0.001	1.56	1.47–1.65	<0.001
**Connective tissue disease**	1.06	1.02–1.10	0.001	1.09	1.04–1.15	0.001	1.04	0.99–1.10	0.116
**Malignancy**	1.38	1.34–1.42	<0.001	1.47	1.41–1.53	<0.001	1.27	1.21–1.34	<0.001
**Polycystic kidney disease**	0.92	0.83–1.01	0.076	0.91	0.82–1.00	0.061	0.99	0.75–1.29	0.928

Multiple Regression: adjusted the variables listed in Table 1.

## Discussion

Our results showed that the incidence of peritonitis was low in HD patients, occurring in only 1.16% of HD patients over 13-year study period. Female gender, liver cirrhosis and PKD are significant risk factors for developing peritonitis in both non-diabetic and diabetic HD patients. Importantly, our data supported our hypotheses that, like PD patients, peritonitis was associated with a significantly increased risk of subsequent mortality in HD patients.

Liver cirrhosis was one of the most significant risk factors for peritonitis in this study (adjusted HR = 3.21, 95% CI = 2.50–4.12 in non-DM group; adjusted HR = 3.08, 95% CI = 2.12–4.49 in DM group). In both groups, cirrhotic HD patients had more than 3 times increased risk for peritonitis compared with non-cirrhotic HD patients. In our study, there were 4,347 (5.4%) patients with liver cirrhosis at the initiation of dialysis. The prevalence is much higher than that reported in western countries, where the estimate is only 1~2% in different studies [17, 18]. This finding is not surprising, because Taiwan is a hyperendemic area of hepatitis B and C virus infections [19]. Patients with advanced stage liver cirrhosis tend to develop spontaneous bacterial peritonitis because of increased bacterial translocation from the gut and impaired immune systems [20]. Therefore, it is conceivable that dialysis patients with advanced liver cirrhosis should also be potentially susceptible to this complication. However, there is a striking paucity of reports about this association in HD patients. An alternative explanation is that liver cirrhosis is also characterized by disturbances in both innate and adaptive immune systems [21–23], which make them more vulnerable to certain infections. To our knowledge, this is the first study to report that cirrhotic HD patients have a greater risk of peritonitis than non-cirrhotic HD patients. Intriguingly, two small studies demonstrated that the incidence of peritonitis was similar or slightly higher in cirrhotic PD patients [24, 25]. Hypotheses in this area need to be tested in further study.

Female gender was another significant risk factor of developing peritonitis. This finding was consistent with some previous studies in the PD population [16, 26]. Perez et al analyzed 693 episodes of peritonitis in PD patients and demonstrated that the risk of experiencing at least one episode of severe peritonitis was 2 times higher among women (RR = 2.03, 95% CI = 1.29–3.16, P = 0.002) [16]. We are not sure of the mechanism for this phenomenon, but it is possible that the female genitourinary tract may be a potential source of yeast and other microorganisms, leading to higher risk of peritonitis.

Another significant risk factor for peritonitis was PKD (adjusted HR = 1.60, 95% CI = 1.02–2.50 in non-DM group; adjusted HR = 4.06, 95% CI = 1.30–12.72 in DM group). Indeed, patients with PKD face an increased risk of renal cyst infection [27]. It was estimated that 30–50% of patients with ADPKD experience renal cyst infections at least once during their lifetime, and age, female gender and recent instrumentation of the urinary tract pose a higher risk [28, 29]. Although the clinical outcome of renal cyst infection is generally good, there are some reports of intraperitoneal rupture of infected renal or liver cysts causing severe peritonitis [30, 31].

In our study, we observe a conflicting result that DM is associated with a decreased risk of peritonitis in HD patients. Part of this unexpected observation may be explained by the fact that diabetic patients may have died over the first few years of dialysis because of cardiovascular event, possibly too early to manifest peritonitis. Indeed, the median timefrom initiation of dialysis to death was shorter in diabetic HD patients than non-diabetic HD patients (2.5 vs 4.0 years). Another possibility is that some diabetic HD patients might have deteriorated to severe sepsis or death before a definite diagnosis of peritonitis, thereby potentially misclassification to severe sepsis without definite focus and leading to underestimate this rare event. Further studies are warranted to better investigate this unexpected association.

In our study, age, myocardial infarction, stroke, heart failure, atrial fibrillation, liver cirrhosis and malignancy were all significantly related to the increase of mortality risk in HD patients. Although these factors are well-established risk factors for mortality as proven in previous literature [32–34], we reported the novel finding that peritonitis had strong prognostic impact of mortality on patients receiving HD (adjusted HR = 2.86, 95% CI = 2.63–3.12, p<0.001). The 1-year-survival after peritonitis was only 38.8%. In contrast, although peritonitis was more common in PD patient, the 1-year-survival after peritonitis could be 70–90% [15, 35]. Notably, among general population, cirrhosis patients who suffered from spontaneous bacterial peritonitis also have increased risk of short- and long-term mortality [36–38]. The survival rates at 1 year following an episode of peritonitis are reported to be 30–40% [38, 39], which is approximate to our current report on HD patients. This increased mortality in cirrhotic patients has been largely attributed to multiple interrelated complications, such as gastrointestinal bleeding, bacteremia, or hepatorenal syndrome. Because only 11% of our peritonitis patients had liver cirrhosis, we assume that multiple insults cause high mortality in HD patient after incident peritonitis. First, peritonitis patients have more comorbidities such as cirrhosis, connective tissue disease, or malignancy. Higher mortality might be related principally to these underlying diseases. Second, peritonitis in HD patients may present with more aggressive forms of peritonitis, which might be more accompanied by septic shock or multi-organ failure. Third, since peritonitis is rare in HD group, incident peritonitis probably suggests impaired immunity or performance status. Further work is needed to explore these possibilities and investigate effective interventions to avoid such events.

There were several limitations of our study. First, the study method was a longitudinal observational study. We could only state the association but not the causality of peritonitis and mortality, and the results might be confounded by diseases that were not calculated in the statistical analysis. Second, we used diagnostic codes to define peritonitis, but there was no detailed description of the cause of peritonitis. We were unable to evaluate etiologic microorganism, the severity, treatment and direct sequela of peritonitis events. Although primary and secondary peritonitis had different pathophysiology, they might both indicate poor outcome. Furthermore, the primary objective of this study was to offer a preliminary assessment of risk for HD patients and to heighten awareness among clinicians about the significance of peritonitis as a cause of poor outcome. Third, the comorbid variables were based on ICD-9-CM diagnostic codes and prescription. We might neglect patients on diet control or those who refused medications. Some information including patient performance status, dialysis prescription and clearance, fasting plasma glucose, glycated hemoglobin, ambulatory blood pressure, or medication compliance were not available. Therefore, our data may reflect inadequate adjustments for these known risk factors.

## Conclusion

Although peritonitis is a rare condition in HD patients, it is associated with poor outcome. Female gender, liver cirrhosis and PKD were three of the most significant factors associated with peritonitis in our study. Additional studies are needed to explore the pathogenesis of each risk factor in HD patients and to investigate effective intervention to prevent this unwanted complication.
